# Ultraviolet-nanoimprinted packaged metasurface thermal emitters for infrared CO_2_ sensing

**DOI:** 10.1088/1468-6996/16/3/035005

**Published:** 2015-05-20

**Authors:** Hideki T Miyazaki, Takeshi Kasaya, Hirotaka Oosato, Yoshimasa Sugimoto, Bongseok Choi, Masanobu Iwanaga, Kazuaki Sakoda

**Affiliations:** 1 National Institute for Materials Science, 1-2-1 Sengen, Tsukuba, Ibaraki 305-0047, Japan; 2 University of Tsukuba, 1-1-1 Tennodai, Tsukuba, Ibaraki 305-8577, Japan

**Keywords:** metamaterials, metasurfaces, thermal emission, infrared emitters

## Abstract

Packaged dual-band metasurface thermal emitters integrated with a resistive membrane heater were manufactured by ultraviolet (UV) nanoimprint lithography followed by monolayer lift-off based on a soluble UV resist, which is mass-producible and cost-effective. The emitters were applied to infrared CO_2_ sensing. In this planar Au/Al_2_O_3_/Au metasurface emitter, orthogonal rectangular Au patches are arrayed alternately and exhibit nearly perfect blackbody emission at 4.26 and 3.95 *μ*m necessary for CO_2_ monitoring at the electric power reduced by 31%. The results demonstrate that metasurface infrared thermal emitters are almost ready for commercialization.

## 1. Introduction

Thermal emission control by various surface nanostructures has been demonstrated [1–6]. Thermally emissive surfaces based on stacked metal/insulator/metal (MIM) structures [7] are attracting particular attention because of their manufacturability, high controllability of thermal emission [8, 9], and significance as metamaterials or metasurfaces [10, 11]. In our previous work [12], we developed a metasurface infrared thermal emitter that exhibits nearly perfect blackbody emission at two wavelengths necessary for CO_2_ sensing while suppressing un-necessary radiation. The emitter was integrated with a resistive heater and we demonstrated the reduction in the electric power compared with a conventional blackbody emitter. This showed that metasurface thermal emitters could contribute to practical applications; however, there were problems that needed to be overcome. The metasurface was fabricated by electron-beam lithography (EBL) with a low throughput, and is impractical for mass-production. In addition, the emitter was incompatible with conventional optoelectronic devices.

In this paper, we demonstrate that dual-band metasurface thermal emitters integrated with a membrane heater can be manufactured by the cost-effective UV-nanoimprint lithography (NIL) technique, which is suitable for mass-production. The use of a soluble UV resist enabled simple fabrication by a monolayer lift-off process. The peak wavelengths were finely tuned by atomic layer deposition (ALD). Moreover, the emitter chips were assembled in standard packages and used for CO_2_ concentration measurement. These results demonstrate that metasurface infrared thermal emitters are now commercially viable.

## 2. Structure design

**Figure 1.**
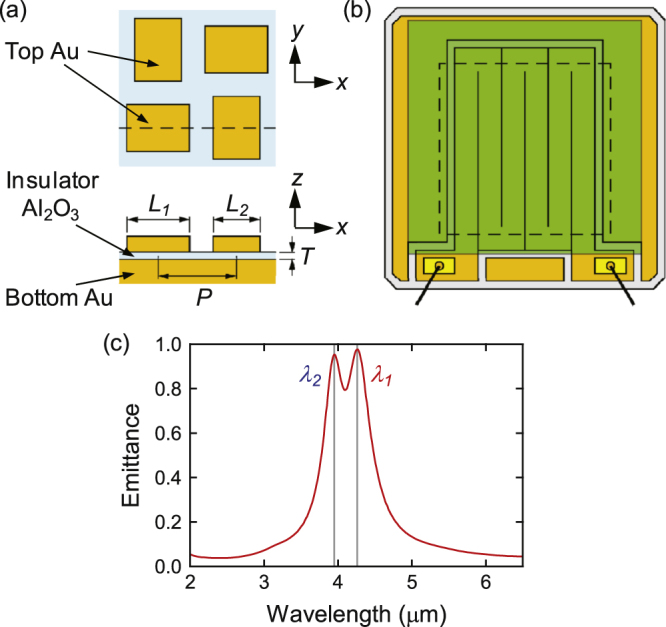
(a) Structure of the dual-band metasurface thermal emitter. The lower panel shows the cross section at the broken line. (b) Overall view of the emitter chip. The metasurface shown in (a) is formed in the green area (3.0 mm square), and the area surrounded by the broken line is supported by a thin membrane. (c) The emittance spectrum in the vertical direction (*z*-axis) for the optimum design for both *x* and *y* polarizations.

The structure and emittance spectrum of the metasurface emitter is shown in figure 1 [12]. On the Al_2_O_3_ film with a thickness *T* on the bottom Au layer, orthogonal rectangular Au patches 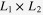 in size are alternately arrayed with a period *P*, where *T* = 50 nm, *L*_1_ = 930 nm, *L*_2_ = 850 nm, and *P* = 1.5 *μ*m. This structure exhibits nearly perfect blackbody emission at two wavelengths of 4.26 and 3.95 *μ*m (referred as *λ*_1_ and *λ*_2_ hereinafter) which are typical wavelengths for CO_2_ sensing, while minimizing radiation at other wavelengths. The spectrum was obtained by rigorous coupled wave analysis (Synopsys, DiffractMOD) based on the reported dielectric constant of Au [13]. The refractive index of Al_2_O_3_ was set at 1.52 [12]. Because the structure does not change after a 90° rotation around the *z*-axis, there is no polarization dependence. In addition, as the emission peak is based on the plasmon resonance in a sub-wavelength-sized structure, the angle dependence is also small.

This surface structure is formed on a meander resistive heater made of Au, as shown in figure 1(b). This heater also functions as the bottom Au layer of the metasurface. The emitter chip is 3.4 mm square and its central 2.1 mm square area is supported by a 500 nm thick amorphous SiN membrane for thermal isolation and thermal mass minimization. The blank space outside the heater is also covered with Au; this feature is required for successful NIL, as will been discussed later.

## 3. Process design and fabrication

In our previous work [12], we fabricated the metasurface by EBL and lift-off. In this work, EBL is replaced with UV–NIL, which is a high throughput technique [14]. Because UV–NIL is a room-temperature process, it takes a shorter time than thermal NIL, in which the temperature is raised and then lowered [15]. However, UV–NIL has a serious drawback: incompatibility with lift-off processes. Lithography-based metal patterning can be achieved through dry etching or lift-off processes. In dry etching, a metal film (and sometimes also a hard mask material) is deposited on the substrate and the resist mask pattern is formed on it. Then, anisotropic dry etching is performed until the metal film is perforated. In contrast, in the lift-off process, a resist pattern is directly formed on the substrate and then a metal film is evaporated on it. The resist is then dissolved in a solvent and the metal mask on the resist is also removed. Because the dry etching process requires accurate depth control, care about the redeposition of materials, and the removal of the mask, the lift-off technique is much simpler and more reliable, and thus suitable for mass-production. Nonetheless, UV resins for UV–NIL are generally insoluble in organic solvents after photocuring. Therefore, lift-off processes for UV–NIL conventionally required bilayer resists including a soluble underlayer and two-step dry etching [16]. However, the complexity of this process negates the advantage of lift-off techniques. Here we employ a soluble UV resist, which was recently developed [17]. This new-generation UV resist enables lift-off with a monolayer resist and preserve the simplicity of the lift-off process.

**Figure 2.**
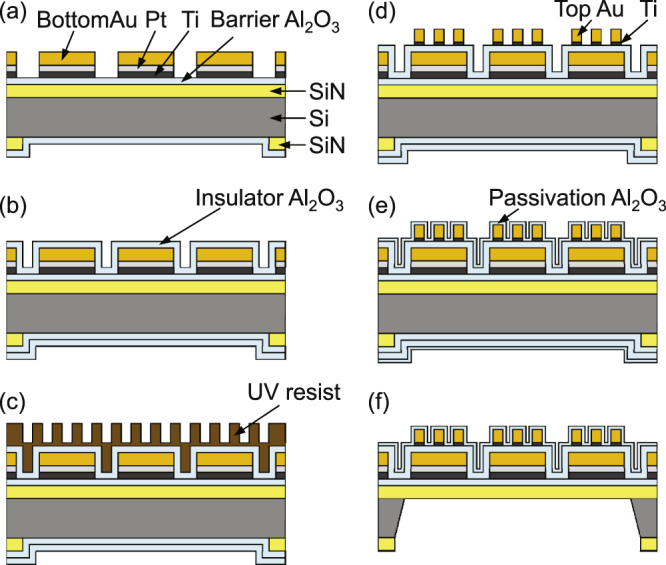
Fabrication process for the metasurface thermal emitter. (a) SiN windows are opened by photolithography and dry etching on the back, the barrier Al_2_O_3_ layer (50 nm) is deposited, the heater is patterned by photolithography, and the Ti (50 nm), Pt (50 nm), and bottom Au (100 nm) films are lifted off. (b) The insulator Al_2_O_3_ layer with a thickness of *T* is deposited. (c) The soluble UV resist is spin-coated. The mold is pressed against the substrate at a pressure of 1.8 MPa for 5 min, then it is irradiated with UV light (365 nm) with a power density of 37 mW cm^−2^ for 10 s and 33 mW cm^−2^ for 60 s. (d) The residual resist is removed by reactive ion etching with O_2_ and N_2_ gases, and then the Ti (3 nm) and top Au (100 nm) layers are evaporated and lifted off. The Ti layer is thicker than that in our previous work to ensure adhesion of the Au patches during the lift-off process. (e) The passivation Al_2_O_3_ layer is deposited with a precisely predetermined thickness according to the absorption spectrum. After steps (b) and (e), photolithography and buffered HF etching are used to open Al_2_O_3_ windows at the electrode areas. (f) The Si substrate is anisotropically etched with KOH (8 mol L^−1^, 82–88 °C) from the back to form the membrane and the cutting slots.

The fabrication steps for the metasurface thermal emitter are shown in figure 2. Both sides of a 30 mm square 0.38 mm thick Si substrate are coated with a 500 nm thick amorphous SiN film, of which the composition is adjusted to be Si-rich to impose a suitable tensile stress. On this substrate, 36 emitter chips 3.4 mm square are arranged in a 6 × 6 matrix. Firstly, SiN windows for the membranes and cutting slots are opened on the back of the substrate (figure 2(a)). After ALD of the first Al_2_O_3_ film, the heater patterns are formed from Ti, Pt, and Au. The Al_2_O_3_ and Pt layers prevent the diffusion of Si atoms into Au and the Ti layer enhances the adhesion between them. Then, the second Al_2_O_3_ layer for the metasurface insulator layer is deposited (figure 2(b)). The process so far is similar to our previous work [12].

We used a micropattern imprinting machine (ST50, Toshiba Machine) for UV–NIL [18, 19]. On a 30 mm square 1 mm thick quartz mold, 250 nm high rectangular mesas corresponding to the Au patches are arrayed. The mold is cleaned with piranha solution and vacuum UV light [20], and then it is coated with a release agent (SAMLAY-A, Nippon Soda). On the heater side of the substrate, a soluble UV resist (NIAC705, Daicel) was spun and prebaked. The resist thickness on the heater pattern is 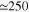 nm. The mold is pressed against the substrate and then UV light is shone through the mold (figure 2(c)). After removing the residual resist layer at the bottom with a thickness of 10–20 nm by dry etching, Ti and Au layers are evaporated and lifted off with *N*-methyl-2-pyrrolidone (figure 2(d)). The third Al_2_O_3_ layer for surface protection (passivation) is deposited by ALD (figure 2(e)), and finally the back is etched with KOH (figure 2(f)).

In NIL, fine adjustment of the patterns is difficult once the mold is prepared. The Au patches fabricated using the mold prepared for obtaining the rectangular patches in figure 1(a) typically had dimensions of *L*_1_ = 1000 nm and *L*_2_ = 920 nm, which was larger than expected. However, an advantage of the MIM-scheme metasurface is the tunability of the optical properties by other parameters. First, by varying the thickness of the metasurface insulator layer, *T*, the resonance wavelength can be tuned through changing the wave vector of the surface plasmon mode [21]; a thicker layer produces a shorter wavelength. Second, the thickness of the passivation layer also affects the resonance; a thicker passivation layer gives longer wavelength [22]. The wavelengths were initially adjusted at the shorter sides with a thick insulator layer, and then finely tuned by the passivation layer with a thickness precision of 0.1 nm.

**Figure 3.**
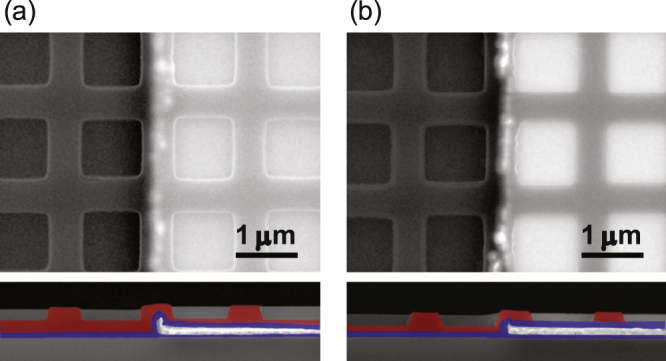
Scanning electron micrographs of the results of UV–NIL on the heater pattern. The upper and lower panels show the top and cross-sectional views, respectively. In each panel, the heater pattern is seen on the right-hand side. The cross sections of the resist and Al_2_O_3_ are colored red and blue, respectively, for clarity. (a) Before and (b) after the residual layer removal.

In most realistic devices, the overlay of multiple patterns is necessary, as in our heater-integrated metasurface emitters. However, there are few reports of NIL on patterned substrates [23]. In the early stage of our study, the residual resist layer on the heater pattern was not uniform; the residual layer was thick at the center of the heater and thin at the edges. The resonance wavelengths of the resultant metasurface were distributed depending on the position in the heater; the wavelengths were short at the center and long at the edges. This is because the heater surface is higher than the blank area. The substrate is deformed during NIL [15], and the depth of the imprinted patterns becomes inhomogeneous. Therefore, in this work, the blank area outside the heater pattern was also coated with the Ti, Pt, and Au layers to adjust the surface levels (figure 1(b)), which drastically improved the optical uniformity. Figure 3 shows the results of the UV–NIL on the heater pattern. In the blank area outside the heater, the bottom of the resist does not reach the substrate after the dry etching. Therefore, the metasurface is patterned only on the heater.

**Figure 4.**
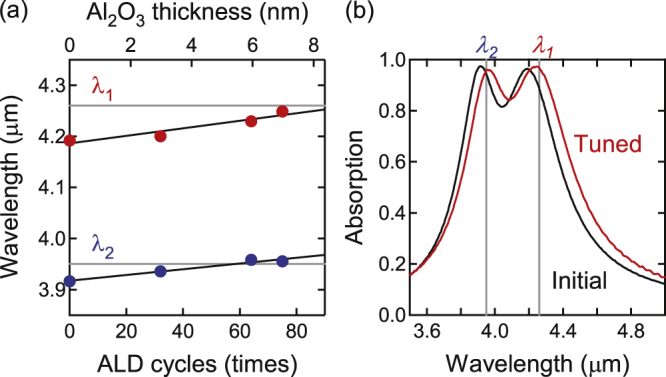
Tuning of the resonant wavelengths for *p* polarization by the passivation Al_2_O_3_ layer. (a) Change in the resonant wavelengths (absorption peak positions) as functions of the passivation Al_2_O_3_ film thickness. In this example, the wavelengths converged at the targets at 75 cycles of ALD. (b) Initial and tuned absorption spectra. The maximum absorption (emittance) after tuning is 0.97. The absorption spectra were measured at 26° from the *z*-axis.

The thickness of the insulator Al_2_O_3_ layer was set at *T* = 81 nm so that wavelengths shorter than the target values were obtained. The passivation Al_2_O_3_ layer thickness was precisely determined according to the measured absorption spectrum. The relationship between the wavelengths and the thickness of the passivation Al_2_O_3_ layer is shown in figure 4. Because independent tuning of *λ*_1_ and *λ*_2_ is not possible by the Al_2_O_3_ thicknesses, we equalized the deviations from the targets at both peaks.

**Figure 5.**
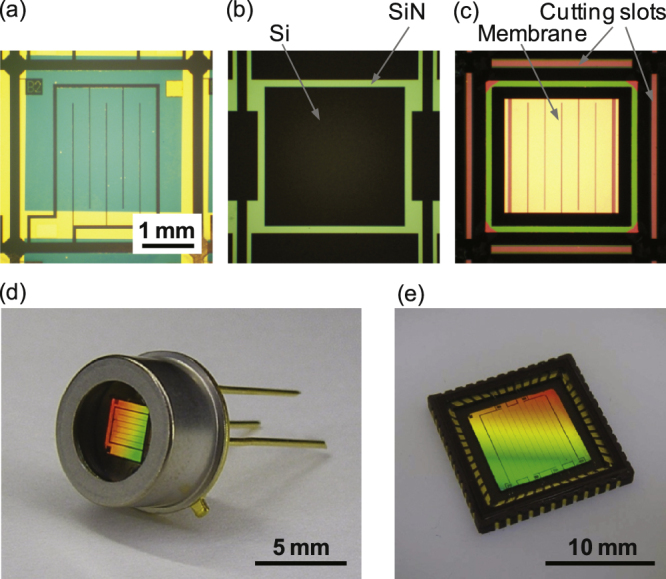
Emitters during fabrication. (a) After the lift-off of the top Au layer. The mold and the substrate are positioned manually with a precision of about ±250 *μ*m. (b) SiN windows on the back, which result in the pattern in (c) after anisotropic etching by KOH. The back of the heater pattern is in the square area at the center. The cutting slots are visible along the four sides of the frame and the chip is supported with four bridges at the corners. The scales in (a)–(c) are the same. (d) A TO-5 packaged metasurface emitter. Emission area: 1.9 × 2.1 mm. Sapphire window thickness: 0.25 mm. (e) A C-QFN (LCC) packaged metasurface emitter. Emission area: 6.3 × 6.6 mm. Sapphire window thickness: 0.30 mm. Chip size: 8.6 × 8.6 mm.

Figure 5 shows a chip during fabrication and the completed emitters. After the KOH etching, the chips are separated by breaking the bridges at four corners (figure 5(c)). The chip is mounted on a TO-5^3^ 3TO: Transistor Outline. C-QFN: Ceramic Quad Flat No Lead Package. LCC: Ceramic Leadless Chip Carrier. stem with epoxy, wire bonded, and hermetically sealed with a cap with a sapphire window (figure 5(d)). The significance of the NIL technique is that a large device can also be fabricated in a short time. In this study, a mold for 10 mm square areas was also prepared and large emitters with a C-QFN(LCC) (see footnote 3) package were also manufactured (figure 5(e)). As the thermal emission power is proportional to the area, a large emitter is necessary for generating large optical power.

## 4. Optical properties

**Figure 6.**
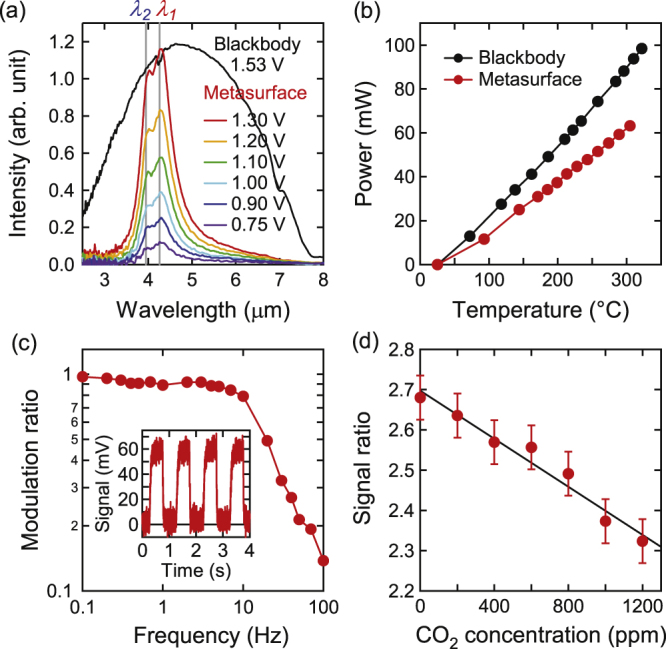
(a) Emission spectra for various voltages for *s* polarization observed from the direction at 23° from the *z*-axis. A spectrum for an equivalent blackbody at the same temperature (305 °C) as the metasurface at 1.30 V is also plotted. The spectra are corrected for the spectrometer response. (b) Relationship between input power and temperature for the metasurface and blackbody emitters. (c) Frequency response of the modulation ratio at *λ*_1_. The voltage amplitude is adjusted so that the maximum temperature is 300 °C at *f* = 1.0 Hz, and the infrared waveform is shown in the inset. The duty ratio of the rectangular modulation is 50%. (d) Signal ratio of *λ*_1_ to *λ*_2_ as a function of CO_2_ concentration. *f* = 1.0 Hz (inset in (c)). The error bars represent the standard deviation for numbers of measurements. The signal-to-noise ratio is low, because the emitter and detector face each other at a distance of 61 mm with no optics to enhance the efficiency.

The properties of the TO-5 emitter in figure 5(d) are shown in figure 6. To avoid thermal loss due to air convection, the thermal emitter should be placed in a vacuum. However, facilities for vacuum encapsulation were not available in this study. Therefore, by evacuating from a 1 mm diameter hole at the bottom of the package, the optical properties in a vacuum (0.2 Pa) were investigated. The fundamental characteristics are very similar to those in the previous report [12], because the resultant emitter is equivalent to the previous work, except for the frame structure.

Figure 6(a) displays the emission spectra of the metasurface emitter at various voltages. The temperature at the maximum voltage of 1.30 V was 305 °C. Figure 6(a) also shows the emission of a blackbody emitter at 305 °C, which required 1.53 V. Our previous report [12] provides details of the determination of the emitter temperature and the preparation of the reference blackbody emitter. Figure 6(a) demonstrates that the metasurface emitter exhibits the same magnitude of radiation at the two wavelengths as a conventional blackbody emitter, at a lower voltage (lower power). The lower output at a longer wavelength region (≥6 *μ*m) than in the previous report [12] is because of the sapphire window.

Figure 6(b) shows the relationship between the input power and the emitter temperature. The metasuface decreases the power required to reach the same temperature, which exhibits the same optical power at the two wavelengths, by 31%. The reduced input power roughly agrees with the estimated decreased emission power. The emission power from the metasurface at 300 °C is estimated to be 3.4 mW; thus, most of the input power (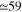 mW) is not used, mainly due to the conduction loss from the membrane to the frame.

In CO_2_ sensors, the emitter power is modulated and the transmitted infrared power is sensitively measured by synchronous detection. The relationship between the modulation frequency, *f*, and the modulation ratio at *λ*_1_ of the metasurface emitter is displayed in figure 6(c). A fast HgCdTe infrared photodetector (VIGO, PVI-2TE-10.6, cut-off frequency: 300 kHz) and a bandpass filter (center: 4.26 *μ*m, width: 0.18 *μ*m) were used. The modulation ratio is defined as 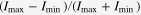, where *I*_max_ and *I*_min_ are the maximum and minimum signal of the photodetector, respectively. The raw signal at *f* = 1 Hz is also shown. The cutoff frequency at −3 dB is *f* = 20 Hz, which is sufficient for CO_2_ sensors.

However, this result suggests a serious limitation in thermal emitters. If the conduction loss is minimized, the heat escape channel is reduced, which degrades the frequency response. Consequently, there is a tradeoff between the power efficiency and the frequency response. To overcome this limit, a modulation mechanism other than direct temperature control must be incorporated. Recently, high-frequency electrical modulation of thermal emission in a photonic-crystal thermal emitter based on intersubband transitions was reported [24]. Similarly, in metasurface thermal emitters, dynamic control of the emittance is of particular importance [25].

Finally, a CO_2_ concentration sensor based on the TO-5-packaged metasurface emitter was constructed. The optical system was similar to that in figure 5(a) of our previous report [12], although the input electric power was directly modulated instead of using a chopper. The emitter and a dual-channel pyroelectric detector (InfraTec, LIM-222-DH) were placed opposite each other, sandwiching a gas cell initially purged with N_2_ gas at 1 atm. As CO_2_ gas was introduced into the cell, the signal ratio of *λ*_1_ to *λ*_2_ linearly decreased (figure 6(d)) at a rate of −4.4% at 400 ppm (typical air). This result demonstrates that our mass-producible dual-band metasurface emitter can be used for CO_2_ sensing.

## 5. Conclusions

In summary, commercially viable metasurface infrared thermal emitters were demonstrated. Dual-band, polarization- and angle-independent metasurface thermal emitters integrated with a resistive membrane heater for CO_2_ sensors were manufactured by a UV–NIL and monolayer lift-off process suitable for mass-production. The use of a soluble UV resist was the key to the simple, practical manufacturing process. The peak wavelengths were accurately adjusted by ALD. Furthermore, hermetically packaged emitters compatible with conventional optoelectronic devices were fabricated and used for measuring CO_2_ concentration. Although further reduction in the thermal loss by improving the membrane design is necessary, the metasurface infrared thermal emitters are almost ready to go to market. The next challenge lies in the development of dynamic modulation mechanisms of the metasurface emitters.
